# Crystal structure and Hirshfeld surface analysis of a new polymorph of chlorido­bis­(1,10-phenan­throline-κ^2^*N*,*N*′)copper(II) perchlorate

**DOI:** 10.1107/S2056989025000209

**Published:** 2025-01-14

**Authors:** Maksym O. Plutenko, Oleksandr S. Vynohradov, Matti Haukka, Irina A. Golenya, Snizhana V. Gaidai

**Affiliations:** a Department of Chemistry National Taras Shevchenko University Volodymyrska Street 64 01601 Kyiv Ukraine; b Department of Chemistry, University of Jyväskylä, PO Box 35, Jyväskylä, FI-40014, Finland; Vienna University of Technology, Austria

**Keywords:** copper, copper(II) complex, crystal structure, 1,10-phenanthroline, Hirshfeld surface analysis

## Abstract

The title salt (systematic name: 2-methyl-4-oxo-3,4-dihydroquinazolin-1-ium chloride), [CuCl(C_12_H_8_N_2_)_2_](ClO_4_), is comprised of a mononuclear complex cation [Cu(phen)_2_Cl]^+^ (phen is 1,10-phenanthroline) and a perchlorate anion, ClO_4_^−^, both with point group symmetry 2. The Cu^II^ atom has a slightly distorted trigonal–bipyramidal coordination environment, defined by a N_4_Cl coordination set with the Cl atom and two N atoms at the equatorial sites. In the crystal, each phen ring is parallel to neighboring phen rings. The resulting significant π–π stacking inter­actions lead to zigzag chains extending parallel to [001]. Hirshfeld surface analysis suggests that the most important contributions to the surface contacts are from H⋯H (32.1%), H⋯C/C⋯H (18.2%), H⋯O/O⋯H (14.6%), H⋯Cl/Cl⋯H (12.7%) and C⋯C (10.6%) inter­actions.

## Abstract 2

Structure analysis of the title compound revealed a second polymorph (space group *C*2/*c*, *Z* = 4) with composition [CuCl(C_12_H_8_N_2_)_2_]^+^ClO_4_^−^. Both the cation and anion in this polymorph exhibit point group symmetry 2.

## 1. Chemical context

1,10-Phenanthroline (phen) is one of the most extensively studied chelating N-heterocyclic ligands. Copper(II) complexes of phen, particularly those exhibiting a coordination environment with coordination number 5, have garnered significant attention due to their diverse biological (Barceló-Oliver *et al.*, 2009 ▸; Pradeep *et al.*, 2014 ▸), redox catalytic (Huang & Batey, 2007 ▸; Liu *et al.*, 2024 ▸), and photochemical (Freitag *et al.*, 2016 ▸) activities. Furthermore, complexes formed between copper and phen (in both 1:1 and 1:2 metal-to-ligand ratios) have been actively investigated as DNA-binding and oxidative DNA-cleaving agents (Bales *et al.*, 2005 ▸; Zhang *et al.*, 2006 ▸; Pradeep *et al.*, 2014 ▸). In this context, structural studies of five-coordinate Cu^II^ complexes based on phen are of considerable inter­est for enhancing the understanding of the geometric features of the CuN_4_*X* chromophore and for elucidating the structure–property relationships of these compounds.
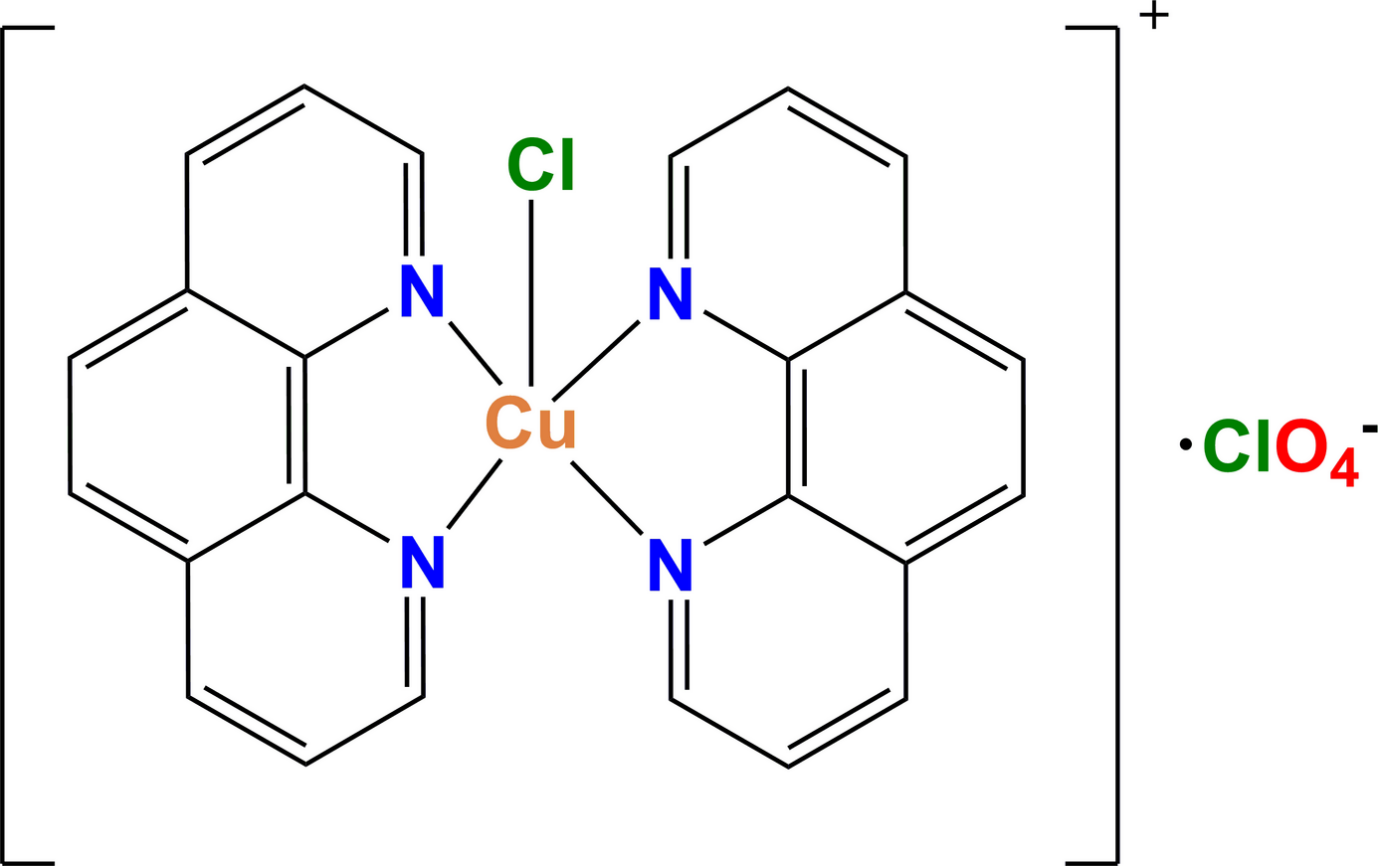


Here, we report on synthesis, crystal structure, and Hirshfeld surface analysis of the compound [CuCl(phen)_2_]ClO_4_, crystallizing as a novel polymorph.

## 2. Structural commentary

**Figure 1:**
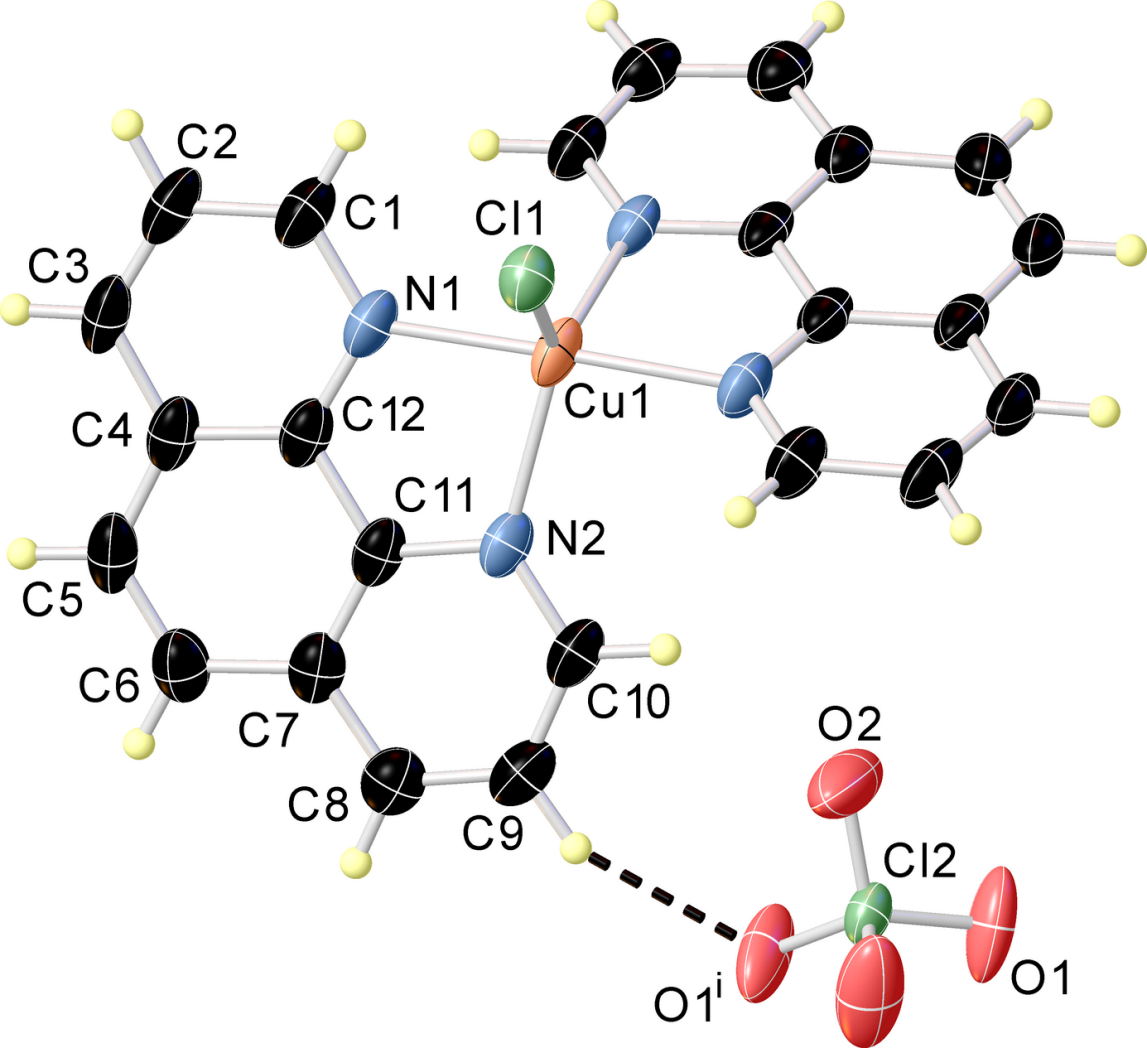
The structures of the mol­ecular entities of the title compound. Displacement ellipsoids are drawn at the 50% probability level. The dashed line represents a C—H⋯O hydrogen bond. [Symmetry code: (i) −*x*, *y*, −*z* + 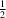.]

The asymmetric unit of the title compound consists of half a [Cu(phen)_2_Cl]^+^ complex cation (point group symmetry 2) and half of a perchlorate anion ClO_4_^−^ (point group symmetry 2). The Cu^II^ ion in the complex cation has a slightly distorted trigonal–bipyramidal coordination environment (τ_5_ = 0.921; Addison *et al.*, 1984 ▸) formed by four nitro­gen atoms from two phen ligands and one Cl^−^ ligand (Fig. 1 ▸). The equatorial plane is defined by atoms Cl1, N1 and N1^i^ whereas the axial positions are occupied by atoms N2 and N2^i^ [symmetry code: (i) −*x*, *y*, −*z* + 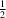]. The Cu—N bond lengths to the equatorial N atoms are shorter by about 0.11 Å in comparison to the axial N atoms (Table 1 ▸). The crystallographically unique phen mol­ecule retains its planarity [maximum deviation from the least-squares plane is 0.034 (3) Å for atom C2] and forms a five-membered [Cu—N—C—C—N] chelate ring due to its bidentate coordination. The dihedral angle between the two phen planes coordinating to Cu1 is 59.55 (6)°. The twist angle between the planes is 48.32 (5)°, the fold angle between the planes is 39.10 (8)°, whereby the twist angle refers to the rotation of one phen plane relative to the other, while the fold angle describes the bending between the planes.

## 3. Supra­molecular features

**Figure 2:**
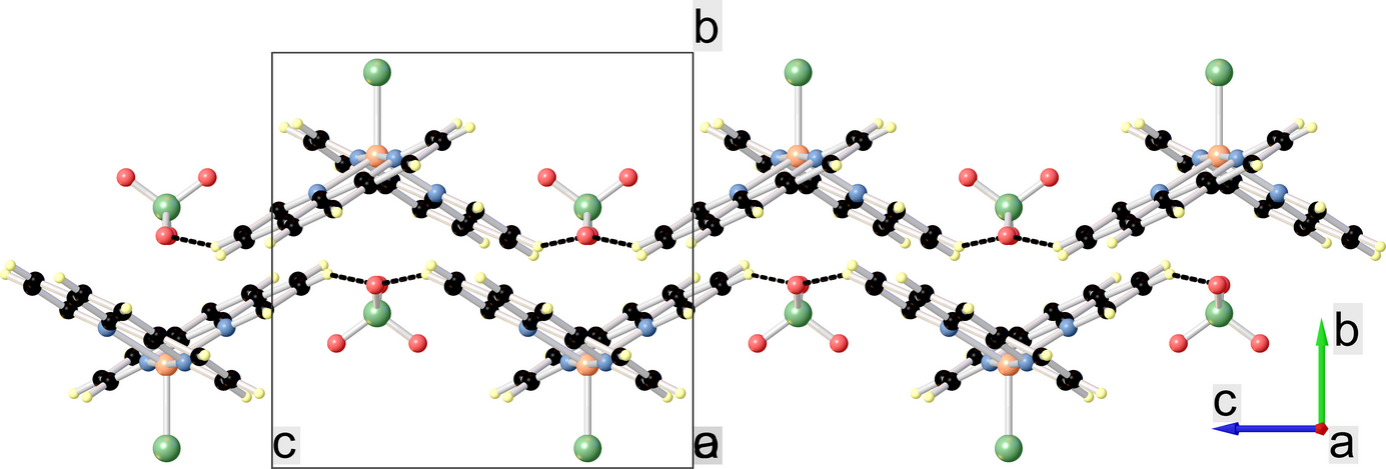
Packing of the mol­ecular components in the title compound in a view along the *a* axis. C—H⋯O hydrogen bonds are shown as black dashed lines.

In the crystal structure of the title compound (Fig. 2 ▸), each phen ring is parallel to its neighboring phen ring. The resulting significant π–π stacking with an inter-planar distance of 3.5085 (16) Å leads to a zigzag chain structure extending parallel to [001]. There are additional weak inter­molecular C—H⋯O hydrogen bonds that link the complex cation to the perchlorate anion. Numerical details of these inter­actions are compiled in Table 2 ▸. The shortest Cu⋯Cu separation within the unit cell is 7.7779 (3) Å.

## 4. Hirshfeld analysis

**Figure 3:**
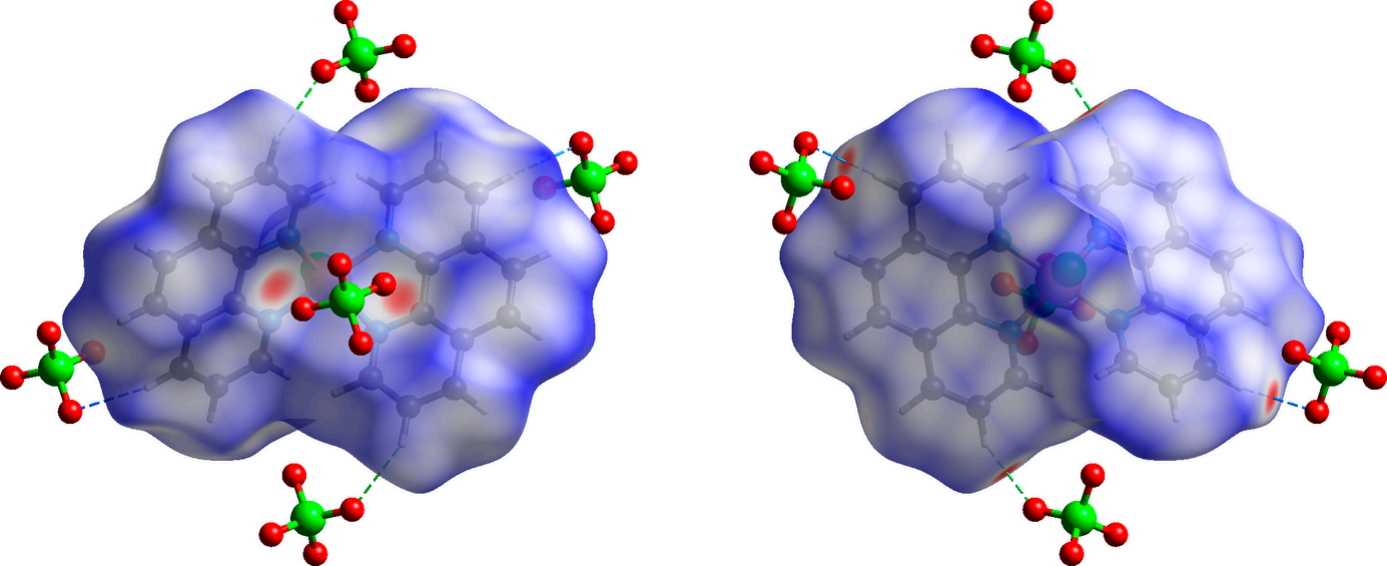
Hirshfeld surface mapped over *d*_norm_ in a projection along the *b* axis (front and back view).

**Figure 4:**
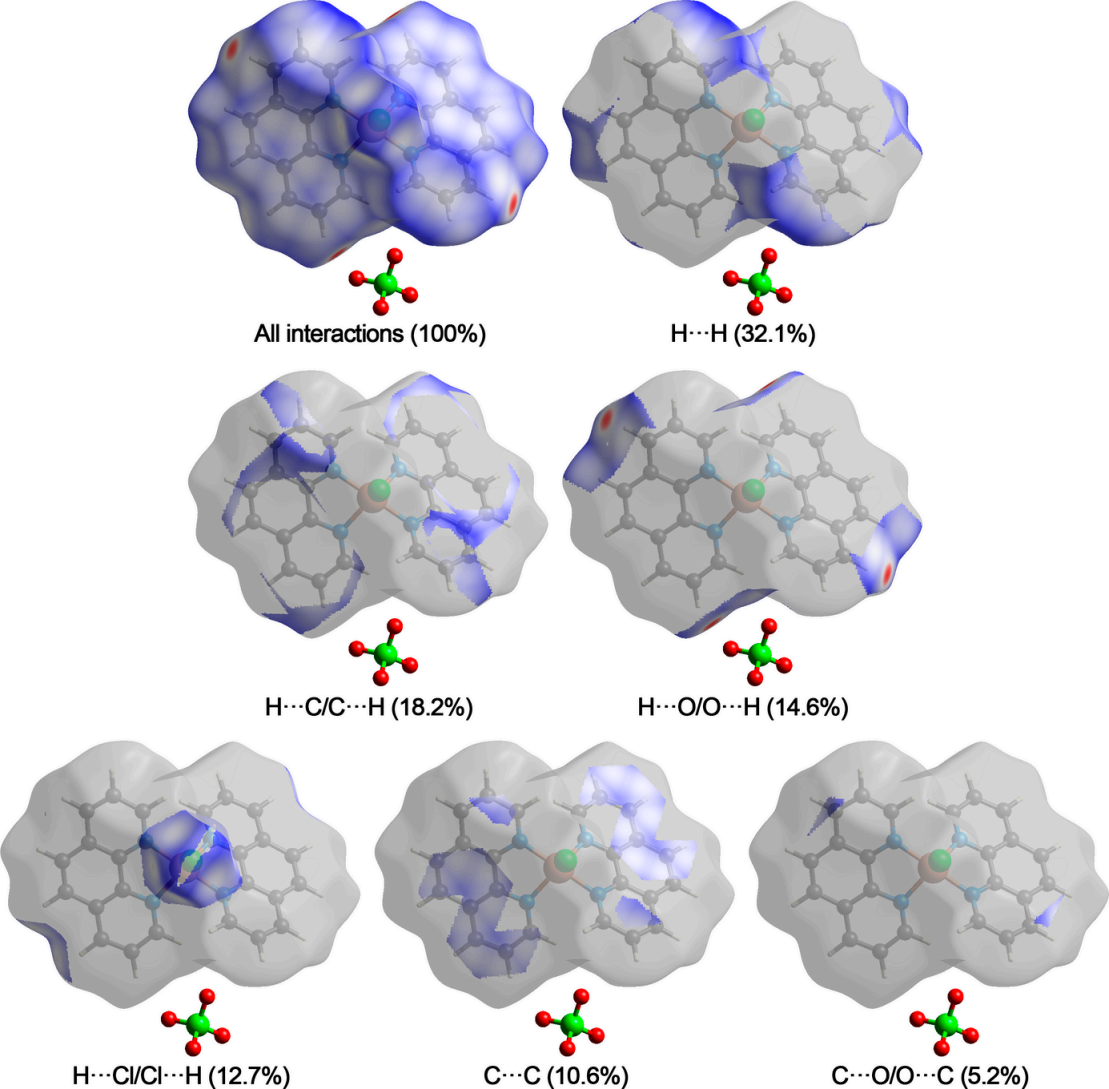
Hirshfeld surface representations with the function *d*_norm_ plotted onto the surface for individual inter­actions.

**Figure 5:**
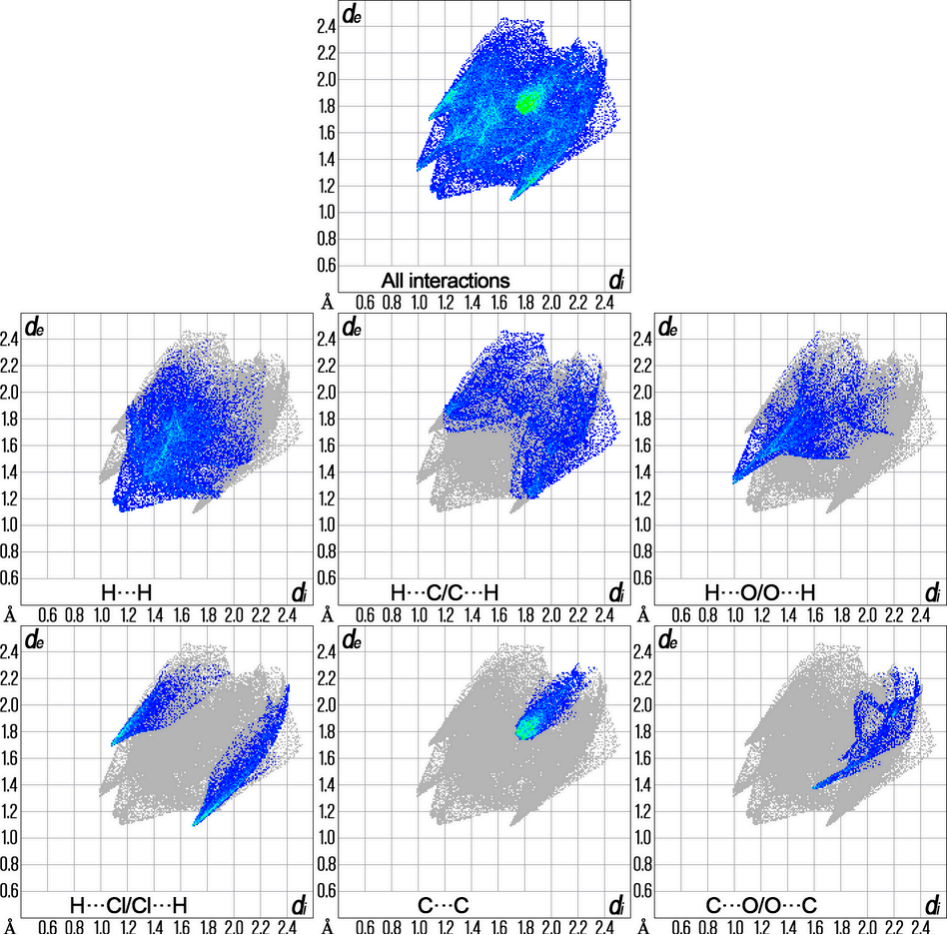
The overall two-dimensional fingerprint plot and those delineated into specified inter­actions.

Hirshfeld surface analysis was performed and the associated two-dimensional fingerprint plots were generated using *CrystalExplorer* (Spackman *et al.*, 2021 ▸). The dark-red spots in Fig. 3 ▸ arise as a result of short inter­atomic contacts and represent negative *d*_norm_ values on the surface, while the other weaker inter­molecular inter­actions appear as light-red spots. The Hirshfeld surfaces mapped over *d*_norm_ are shown individually for the H⋯H, H⋯C/C⋯H, H⋯O/O⋯H, H⋯Cl/Cl⋯H, C⋯C and C⋯O/O⋯C contacts (Fig. 4 ▸). The overall two-dimensional fingerprint plot and those decomposed into individual contacts are given in Fig. 5 ▸. The most significant contributions to the overall crystal packing are from H⋯H (32.1%), H⋯C/C⋯H (18.2%), H⋯O/O⋯H (14.6%), H⋯Cl/Cl⋯H (12.7%) and C⋯C (10.6%) contacts. There are also small contributions from C⋯O/O⋯C (5.2%), H⋯N/N⋯H (2.4%), C⋯N/N⋯C (2.2%), O⋯N/N⋯O (1.8%) and O⋯Cl/Cl⋯O (0.3%) inter­molecular contacts.

In the context of the Hirshfeld surface analysis, qu­anti­tative physical properties for the title compound were obtained, such as mol­ecular volume (460.76 Å^3^), surface area (398.28 Å^2^), globularity (0.724) and asphericity (0.097). The asphericity value for the title compound is nearly zero, indicating an almost isotropic nature. The globularity value, being less than one, points to a slight deviation from a spherical shape.

## 5. Database survey

A search conducted in the Cambridge Structural Database (CSD, version 5.44, updated June 2023; Groom *et al.*, 2016 ▸) identified a total of 118 entries corresponding to compounds containing the [CuCl(phen)_2_]^+^ cation. Among these, five entries specifically pertain to compounds that include both the [CuCl(phen)_2_]^+^ cation and the perchlorate anion. Notably, one structure was identified with the same formula as the title compound, [CuCl(phen)_2_]ClO_4_ (CLPLCU, Boys *et al.*, 1981 ▸; CLPLCU01, Daizhi *et al.*, 2006 ▸), revealing that this compound is at least dimorphic. The other matches have formulas [CuCl(phen)_2_]ClO_4_·0.5H_2_O (ASUCOG, Wei & Yang, 2004 ▸) and [CuCl(phen)_2_]ClO_4_·H_2_O (FUVWUP, Chang *et al.*, 2008 ▸; JATRAA, Crispini *et al.*, 2018 ▸).

CLPLCU and ASUCOG crystallize in space group *P*2_1_/*c*, FUVWUP in *P*2_1_/*n* and JATRAA in *P*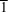. The cell volumes are 4490.3 Å^3^ for ASUCOG (*Z* = 8), 2283.0 Å^3^ for CLPLCU (*Z* = 4), 2335.18 Å^3^ for FUVWUP (*Z* = 4), 1185.88 Å^3^ for JATRAA (*Z* = 2), and 2153.12 Å^3^ for the title compound (*C*2/*c*, Z = 4). Thus, the title compound has the smallest volume per formula unit.

ASUCOG contains two crystallographically unique [CuCl(phen)_2_]^+^ cations, while all other structures contain only one crystallographically unique complex cation. In general, bond lengths and angles of the [CuCl(phen)_2_]^+^ cation are very similar for all described complexes. Cu—N bond lengths lie in the range 1.970–2.135 Å, in good agreement with the title complex, whereas Cu—Cl bond lengths (2.269–2.326 Å) are slightly shorter than in the title compound. The crystal structures of all hydrous compounds (ASUCOG, FUVWUP, JATRAA) include Cu—Cl⋯HOH⋯O—Cl hydrogen-bond motifs.

## 6. Synthesis and crystallization

**Figure 6:**
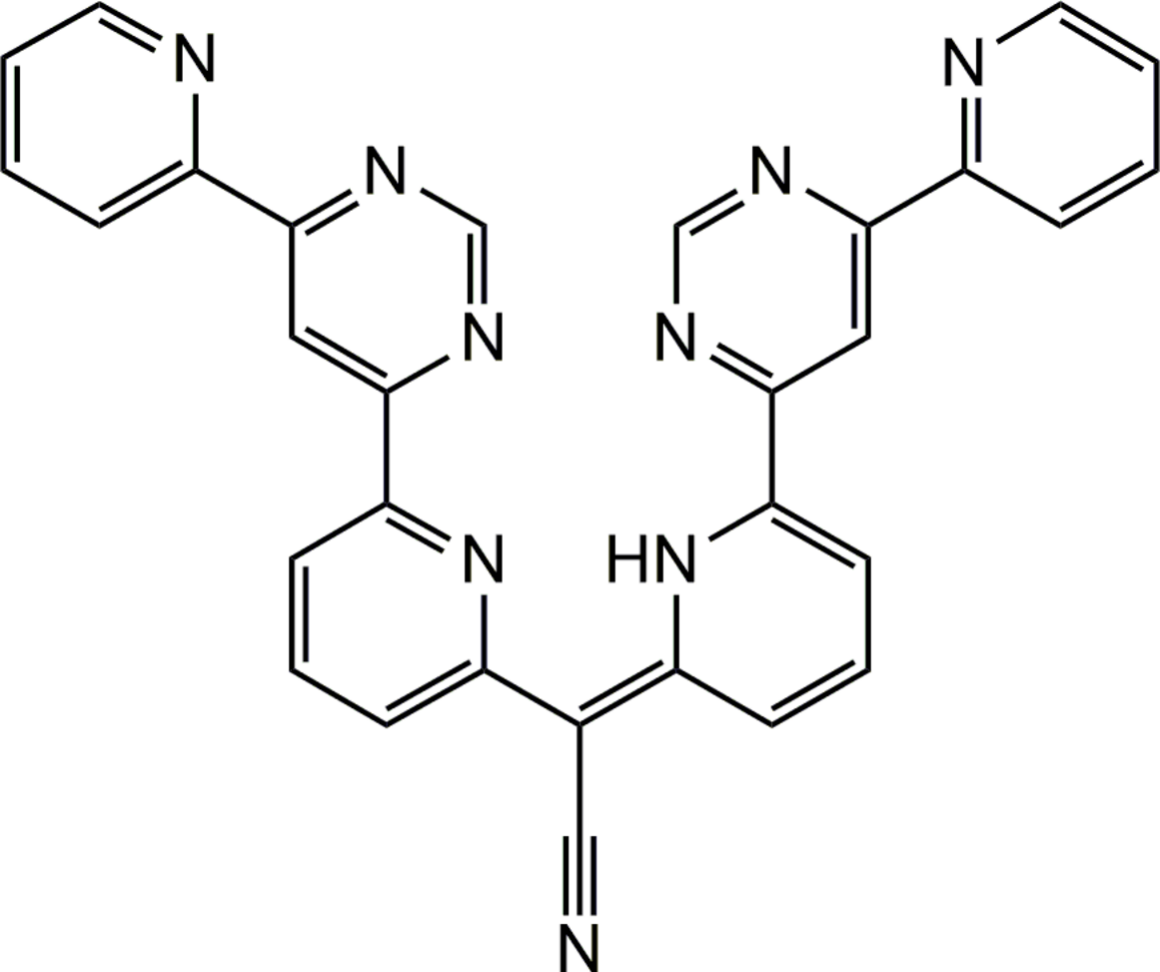
The polypyridyl ligand involved in the synthesis of the title compound.

The title compound was obtained during an attempt to synthesize a trinuclear complex based on the polypyridyl ligand *L*, which includes one tetra­dentate and two bidentate metal-binding sites (Fig. 6 ▸; Fritsky *et al.*, 2003 ▸; Strotmeyer *et al.*, 2003 ▸). Copper(II) chloride dihydrate (0.034 g, 0.2 mmol) dissolved in methanol (5 ml) was added to a solution of 1,10-phenanthroline (0.036 g, 0.2 mmol) in methanol (5 ml). Separately, [Cu(*L*-H)(MeOH)]ClO_4_ (0.070 g, 0.1 mmol), prepared according to Fritsky *et al.* (2001 ▸), was dissolved in a 1:1 (*v*:*v*) methanol–water mixture (10 ml). The two solutions were combined, stirred in air for 30 min. while heated (323 K), then cooled, filtered, and left at room temperature for crystallization. X-ray-quality, small green block-like crystals formed after two weeks. Yield: 0.034 g (61%).

## 7. Refinement

Crystal data, data collection and structure refinement details are summarized in Table 3 ▸. The positions of H atoms were positioned geometrically and refined isotropically using a riding model with C—H = 0.95 Å; *U*_iso_(H) = 1.2*U*_eq_(C).

## Supplementary Material

Crystal structure: contains datablock(s) I. DOI: 10.1107/S2056989025000209/wm5744sup1.cif

Structure factors: contains datablock(s) I. DOI: 10.1107/S2056989025000209/wm5744Isup2.hkl

Supporting information file. DOI: 10.1107/S2056989025000209/wm5744Isup3.cdx

CCDC reference: 2415829

Additional supporting information:  crystallographic information; 3D view; checkCIF report

## Figures and Tables

**Table 1:** Selected bond lengths (Å)

Cu1—N1	1.988 (2)	Cl2—O2	1.418 (3)
Cu1—N2	2.100 (2)	Cl2—O1	1.420 (2)
Cu1—Cl1	2.3402 (12)		

**Table 2:** Hydrogen-bond geometry (Å, °)

*D*—H⋯*A*	*D*—H	H⋯*A*	*D*⋯*A*	*D*—H⋯*A*
C9—H9⋯O1^i^	0.95	2.43	3.253 (4)	145
C3—H3⋯O2^ii^	0.95	2.48	3.368 (5)	157

Symmetry codes: (i) 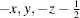; (ii) 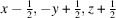.

**Table 3:** Experimental details

Crystal data	
Chemical formula	[CuCl(C_12_H_8_N_2_)_2_](ClO_4_)
*M*_r_	558.85
Crystal system, space group	Monoclinic, *C*2/*c*
Temperature (K)	120
*a*, *b*, *c* (Å)	15.7143 (5), 11.6386 (4), 13.0138 (5)
β (°)	115.227 (2)
*V* (Å^3^)	2153.12 (13)
*Z*	4
Radiation type	Mo *K*α
μ (mm^−1^)	1.31
Crystal size (mm)	0.13 × 0.13 × 0.09

Data collection	
Diffractometer	Nonius KappaCCD
Absorption correction	Multi-scan (*SADABS*; Krause *et al.*, 2015 ▸)
*T*_min_, *T*_max_	0.642, 0.880
No. of measured, independent and observed [*I* > 2σ(*I*)] reflections	21362, 2471, 1947
*R*_int_	0.050
(sin θ/λ)_max_ (Å^−1^)	0.650

Refinement	
*R*[*F*^2^ > 2σ(*F*^2^)], *wR*(*F*^2^), *S*	0.044, 0.105, 1.06
No. of reflections	2471
No. of parameters	160
H-atom treatment	H-atom parameters constrained
Δρ_max_, Δρ_min_ (e Å^−3^)	0.70, −0.57

Computer programs: *COLLECT* (Bruker, 2004 ▸), *DENZO*/*SCALEPACK* (Otwinowski & Minor, 1997 ▸), *SHELXS97* (Sheldrick, 2008 ▸), *SHELXL2019/2* (Sheldrick, 2015 ▸), *OLEX2* (Dolomanov *et al.*, 2009 ▸) and *publCIF* (Westrip, 2010 ▸).

## supplementary crystallographic information

### Chloridobis(1,10-phenanthroline-κ2N,N')copper(II) perchlorate Crystal data

**Table:**

[CuCl(C_12_H_8_N_2_)_2_](ClO_4_)	*F*(000) = 1132
*M**_r_* = 558.85	*D*_x_ = 1.724 Mg m^−^^3^
Monoclinic, *C*2/*c*	Mo *K*α radiation, λ = 0.71073 Å
*a* = 15.7143 (5) Å	Cell parameters from 1037 reflections
*b* = 11.6386 (4) Å	θ = 3.1–27.5°
*c* = 13.0138 (5) Å	µ = 1.31 mm^−^^1^
β = 115.227 (2)°	*T* = 120 K
*V* = 2153.12 (13) Å^3^	Block, green
*Z* = 4	0.13 × 0.13 × 0.09 mm

### Chloridobis(1,10-phenanthroline-κ2N,N')copper(II) perchlorate Data collection

**Table:**

Nonius KappaCCD diffractometer	2471 independent reflections
Radiation source: fine-focus sealed tube	1947 reflections with *I* > 2σ(*I*)
Detector resolution: 9 pixels mm^-1^	*R*_int_ = 0.050
φ scans and ω scans with κ offset	θ_max_ = 27.5°, θ_min_ = 2.3°
Absorption correction: multi-scan (SADABS; Krause *et al.*, 2015)	*h* = −20→20
*T*_min_ = 0.642, *T*_max_ = 0.880	*k* = −15→15
21362 measured reflections	*l* = −16→16

### Chloridobis(1,10-phenanthroline-κ2N,N')copper(II) perchlorate Refinement

**Table:**

Refinement on *F*^2^	0 restraints
Least-squares matrix: full	Hydrogen site location: inferred from neighbouring sites
*R*[*F*^2^ > 2σ(*F*^2^)] = 0.044	H-atom parameters constrained
*wR*(*F*^2^) = 0.105	*w* = 1/[σ^2^(*F*_o_^2^) + (0.0393*P*)^2^ + 5.6416*P*] where *P* = (*F*_o_^2^ + 2*F*_c_^2^)/3
*S* = 1.06	(Δ/σ)_max_ < 0.001
2471 reflections	Δρ_max_ = 0.70 e Å^−^^3^
160 parameters	Δρ_min_ = −0.56 e Å^−^^3^

### Chloridobis(1,10-phenanthroline-κ2N,N')copper(II) perchlorate Special details

**Table:**

Geometry. All esds (except the esd in the dihedral angle between two l.s. planes) are estimated using the full covariance matrix. The cell esds are taken into account individually in the estimation of esds in distances, angles and torsion angles; correlations between esds in cell parameters are only used when they are defined by crystal symmetry. An approximate (isotropic) treatment of cell esds is used for estimating esds involving l.s. planes.

### Chloridobis(1,10-phenanthroline-κ2N,N')copper(II) perchlorate Fractional atomic coordinates and isotropic or equivalent isotropic displacement parameters (Å^2^)

**Table:**

	*x*	*y*	*z*	*U*_iso_*/*U*_eq_	
Cu1	0.000000	0.24858 (4)	0.250000	0.03782 (18)	
Cl1	0.000000	0.04751 (9)	0.250000	0.0380 (3)	
Cl2	0.000000	0.37251 (8)	−0.250000	0.0303 (2)	
O1	0.07641 (18)	0.4406 (2)	−0.2458 (3)	0.0632 (8)	
O2	0.0293 (2)	0.3002 (3)	−0.1533 (3)	0.0819 (10)	
N1	−0.10679 (18)	0.2549 (2)	0.2935 (2)	0.0364 (6)	
N2	−0.09691 (17)	0.33812 (19)	0.1083 (2)	0.0320 (5)	
C1	−0.1099 (2)	0.2086 (3)	0.3852 (3)	0.0430 (7)	
H1	−0.055627	0.170922	0.439318	0.052*	
C2	−0.1913 (2)	0.2140 (3)	0.4040 (3)	0.0438 (8)	
H2	−0.191784	0.179833	0.470096	0.053*	
C3	−0.2694 (2)	0.2679 (3)	0.3281 (3)	0.0399 (7)	
H3	−0.324559	0.271466	0.340803	0.048*	
C4	−0.2683 (2)	0.3186 (2)	0.2303 (2)	0.0349 (7)	
C5	−0.3467 (2)	0.3764 (3)	0.1452 (3)	0.0415 (7)	
H5	−0.403151	0.384501	0.154666	0.050*	
C6	−0.3421 (2)	0.4199 (3)	0.0512 (3)	0.0405 (7)	
H6	−0.395219	0.458075	−0.004311	0.049*	
C7	−0.2578 (2)	0.4090 (2)	0.0343 (3)	0.0343 (6)	
C8	−0.2487 (2)	0.4508 (2)	−0.0620 (3)	0.0382 (7)	
H8	−0.300060	0.487821	−0.121269	0.046*	
C9	−0.1647 (2)	0.4373 (2)	−0.0691 (3)	0.0385 (7)	
H9	−0.157107	0.466637	−0.132784	0.046*	
C10	−0.0908 (2)	0.3806 (2)	0.0169 (3)	0.0359 (7)	
H10	−0.033379	0.371728	0.010016	0.043*	
C11	−0.1797 (2)	0.3536 (2)	0.1167 (2)	0.0308 (6)	
C12	−0.1844 (2)	0.3084 (2)	0.2167 (2)	0.0320 (6)	

### Chloridobis(1,10-phenanthroline-κ2N,N')copper(II) perchlorate Atomic displacement parameters (Å^2^)

**Table:**

	*U*^11^	*U*^22^	*U*^33^	*U*^12^	*U*^13^	*U*^23^
Cu1	0.0417 (3)	0.0452 (3)	0.0423 (3)	0.000	0.0331 (3)	0.000
Cl1	0.0354 (5)	0.0433 (6)	0.0415 (6)	0.000	0.0222 (5)	0.000
Cl2	0.0319 (5)	0.0321 (5)	0.0365 (5)	0.000	0.0239 (4)	0.000
O1	0.0645 (16)	0.0475 (14)	0.112 (2)	−0.0249 (12)	0.0710 (17)	−0.0274 (14)
O2	0.0681 (18)	0.123 (3)	0.0741 (19)	0.0427 (18)	0.0489 (16)	0.0583 (19)
N1	0.0428 (14)	0.0412 (13)	0.0388 (13)	−0.0026 (11)	0.0303 (12)	−0.0019 (11)
N2	0.0399 (13)	0.0307 (12)	0.0373 (13)	−0.0015 (10)	0.0278 (11)	−0.0046 (10)
C1	0.0514 (19)	0.0477 (17)	0.0452 (18)	−0.0009 (15)	0.0352 (16)	0.0023 (14)
C2	0.057 (2)	0.0477 (17)	0.0468 (18)	−0.0089 (15)	0.0414 (17)	−0.0061 (15)
C3	0.0404 (17)	0.0485 (18)	0.0453 (17)	−0.0152 (14)	0.0321 (15)	−0.0160 (14)
C4	0.0384 (16)	0.0372 (15)	0.0397 (16)	−0.0130 (12)	0.0269 (14)	−0.0174 (13)
C5	0.0330 (15)	0.0512 (18)	0.0486 (18)	−0.0122 (13)	0.0254 (14)	−0.0187 (15)
C6	0.0333 (15)	0.0471 (18)	0.0437 (18)	−0.0033 (13)	0.0190 (14)	−0.0113 (14)
C7	0.0367 (15)	0.0327 (14)	0.0389 (16)	−0.0083 (12)	0.0211 (13)	−0.0129 (12)
C8	0.0434 (17)	0.0359 (15)	0.0383 (17)	−0.0038 (13)	0.0202 (14)	−0.0067 (12)
C9	0.0538 (19)	0.0337 (15)	0.0377 (16)	−0.0058 (14)	0.0288 (15)	−0.0025 (13)
C10	0.0455 (17)	0.0347 (15)	0.0403 (16)	−0.0030 (13)	0.0305 (14)	−0.0028 (12)
C11	0.0383 (15)	0.0281 (13)	0.0355 (15)	−0.0089 (11)	0.0248 (13)	−0.0122 (11)
C12	0.0383 (15)	0.0312 (14)	0.0357 (15)	−0.0089 (12)	0.0246 (13)	−0.0116 (12)

### Chloridobis(1,10-phenanthroline-κ2N,N')copper(II) perchlorate Geometric parameters (Å, º)

**Table:**

Cu1—N1	1.988 (2)	C3—C4	1.409 (4)
Cu1—N1^i^	1.988 (2)	C3—H3	0.9500
Cu1—N2^i^	2.100 (2)	C4—C12	1.408 (4)
Cu1—N2	2.100 (2)	C4—C5	1.426 (5)
Cu1—Cl1	2.3402 (12)	C5—C6	1.355 (5)
Cl2—O2^ii^	1.418 (3)	C5—H5	0.9500
Cl2—O2	1.418 (3)	C6—C7	1.438 (4)
Cl2—O1	1.420 (2)	C6—H6	0.9500
Cl2—O1^ii^	1.420 (2)	C7—C11	1.397 (4)
N1—C1	1.330 (4)	C7—C8	1.406 (4)
N1—C12	1.354 (4)	C8—C9	1.370 (4)
N2—C10	1.329 (3)	C8—H8	0.9500
N2—C11	1.363 (3)	C9—C10	1.389 (4)
C1—C2	1.403 (4)	C9—H9	0.9500
C1—H1	0.9500	C10—H10	0.9500
C2—C3	1.358 (5)	C11—C12	1.435 (4)
C2—H2	0.9500		
			
N1—Cu1—N1^i^	175.73 (14)	C2—C3—H3	120.1
N1—Cu1—N2^i^	96.66 (9)	C4—C3—H3	120.1
N1^i^—Cu1—N2^i^	81.20 (9)	C12—C4—C3	116.8 (3)
N1—Cu1—N2	81.20 (9)	C12—C4—C5	119.2 (3)
N1^i^—Cu1—N2	96.66 (9)	C3—C4—C5	124.0 (3)
N2^i^—Cu1—N2	120.50 (12)	C6—C5—C4	121.0 (3)
N1—Cu1—Cl1	92.13 (7)	C6—C5—H5	119.5
N1^i^—Cu1—Cl1	92.13 (7)	C4—C5—H5	119.5
N2^i^—Cu1—Cl1	119.75 (6)	C5—C6—C7	120.9 (3)
N2—Cu1—Cl1	119.75 (6)	C5—C6—H6	119.6
O2^ii^—Cl2—O2	107.1 (3)	C7—C6—H6	119.6
O2^ii^—Cl2—O1	108.37 (15)	C11—C7—C8	117.0 (3)
O2—Cl2—O1	110.36 (19)	C11—C7—C6	119.2 (3)
O2^ii^—Cl2—O1^ii^	110.36 (19)	C8—C7—C6	123.8 (3)
O2—Cl2—O1^ii^	108.37 (15)	C9—C8—C7	119.2 (3)
O1—Cl2—O1^ii^	112.1 (2)	C9—C8—H8	120.4
C1—N1—C12	118.9 (3)	C7—C8—H8	120.4
C1—N1—Cu1	126.8 (2)	C8—C9—C10	119.9 (3)
C12—N1—Cu1	114.29 (18)	C8—C9—H9	120.1
C10—N2—C11	117.2 (3)	C10—C9—H9	120.1
C10—N2—Cu1	132.0 (2)	N2—C10—C9	122.9 (3)
C11—N2—Cu1	110.81 (18)	N2—C10—H10	118.5
N1—C1—C2	121.6 (3)	C9—C10—H10	118.5
N1—C1—H1	119.2	N2—C11—C7	123.8 (2)
C2—C1—H1	119.2	N2—C11—C12	116.4 (3)
C3—C2—C1	120.2 (3)	C7—C11—C12	119.9 (2)
C3—C2—H2	119.9	N1—C12—C4	122.9 (3)
C1—C2—H2	119.9	N1—C12—C11	117.3 (2)
C2—C3—C4	119.7 (3)	C4—C12—C11	119.8 (3)
			
C12—N1—C1—C2	0.0 (5)	C10—N2—C11—C12	−179.2 (2)
Cu1—N1—C1—C2	−176.8 (2)	Cu1—N2—C11—C12	0.4 (3)
N1—C1—C2—C3	−0.3 (5)	C8—C7—C11—N2	−0.2 (4)
C1—C2—C3—C4	−0.1 (5)	C6—C7—C11—N2	179.5 (2)
C2—C3—C4—C12	0.8 (4)	C8—C7—C11—C12	−179.7 (2)
C2—C3—C4—C5	179.5 (3)	C6—C7—C11—C12	0.0 (4)
C12—C4—C5—C6	1.0 (4)	C1—N1—C12—C4	0.8 (4)
C3—C4—C5—C6	−177.7 (3)	Cu1—N1—C12—C4	178.0 (2)
C4—C5—C6—C7	0.0 (5)	C1—N1—C12—C11	−177.7 (3)
C5—C6—C7—C11	−0.5 (4)	Cu1—N1—C12—C11	−0.5 (3)
C5—C6—C7—C8	179.1 (3)	C3—C4—C12—N1	−1.2 (4)
C11—C7—C8—C9	−1.2 (4)	C5—C4—C12—N1	180.0 (3)
C6—C7—C8—C9	179.2 (3)	C3—C4—C12—C11	177.3 (2)
C7—C8—C9—C10	1.5 (4)	C5—C4—C12—C11	−1.5 (4)
C11—N2—C10—C9	−1.0 (4)	N2—C11—C12—N1	0.1 (4)
Cu1—N2—C10—C9	179.5 (2)	C7—C11—C12—N1	179.6 (2)
C8—C9—C10—N2	−0.4 (4)	N2—C11—C12—C4	−178.5 (2)
C10—N2—C11—C7	1.3 (4)	C7—C11—C12—C4	1.0 (4)
Cu1—N2—C11—C7	−179.1 (2)		

Symmetry codes: (i) −*x*, *y*, −*z*+1/2; (ii) −*x*, *y*, −*z*−1/2.

### Chloridobis(1,10-phenanthroline-κ2N,N')copper(II) perchlorate Hydrogen-bond geometry (Å, º)

**Table:**

*D*—H···*A*	*D*—H	H···*A*	*D*···*A*	*D*—H···*A*
C9—H9···O1^ii^	0.95	2.43	3.253 (4)	145
C3—H3···O2^iii^	0.95	2.48	3.368 (5)	157

Symmetry codes: (ii) −*x*, *y*, −*z*−1/2; (iii) *x*−1/2, −*y*+1/2, *z*+1/2.
